# A reproducible infection protocol for entomopathogenic nematodes in the natural host *Plodia interpunctella* larvae

**DOI:** 10.1016/j.mex.2026.103926

**Published:** 2026-04-21

**Authors:** Sreeradha Mallick, George Joseph Chakkalakkal, Taylor Kirby, Juliana Isaac, Vladimir Lažetić, Ioannis Eleftherianos

**Affiliations:** a Entomopathogenic Nematode Lab, School of Biological Sciences, Institute for Global Food Security, Queen’s University Belfast, Belfast BT9 5DL, UK; b Department of Biological Sciences, The George Washington University, WA, DC 20052, USA

**Keywords:** Entomopathogenic nematodes, *Plodia interpunctella*, Host-parasite interactions, Biological control, Nematode pathogenesis

## Abstract

Entomopathogenic nematodes (EPNs) are important for the biological control of insect pests. At the same time, EPNs are excellent research tools for understanding the molecular and functional bases of the insect defense against parasitic nematode infection. Implementing insect models and natural hosts forms an important strategy for characterizing EPN virulence factors and insect anti-nematode immune responses. The Indian meal moth *Plodia interpunctella* is a world-wide insect pest of stored-products and processed food commodities. This insect species is also commonly infected by EPNs, and therefore, it can be used as a natural host to determine how insect pests interact with EPNs during infection. Obtaining this information is critical because it will allow agricultural practitioners to design improved EPN management tactics in the field. Here we describe a protocol for infecting *P. interpunctella* larvae with the EPNs *Steinernema carpocapsae* and *S. hermaphroditum*. The method is outlined below:

• Third instar *P. interpunctella* larvae are infected with infective juveniles of either *S. carpocapsae* or *S. hermaphroditum*. Uninfected control larvae are exposed to sterile water only.
• Insect survival is recorded at regular intervals.
• Survival curves are constructed and results are statistically analyzed to compare the *P. interpunctella* larval mortality against the EPNs.

## Graphical abstract

**Figure:**
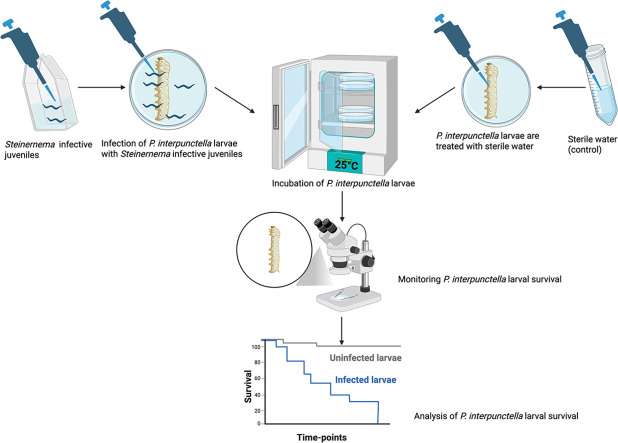

## Specifications table

**Table:** 

**Subject area**	Agricultural and Biological Sciences
**More specific subject area**	Parasitology
**Name of your method**	*Plodia* infection assay
**Name and reference of original method**	N/A
**Resource availability**	N/A

## Background

The Indian meal moth, *Plodia interpunctella* (Hübner, [1813]), is a major insect pest of stored agricultural products. Female moths deposit eggs directly on stored food products, and the larvae burrow inside, causing extensive damage and significant economic loss[1]. Larvae are recognized based on body size and head capsule size. During development, the larvae continuously produce silk webbing spanning over the diet surface, creating a microenvironment [1]. Larval stages cause most damage by penetrating packaging materials. Furthermore, the accumulation of silk webbing, excreta, and cast skin significantly worsens the stored food quality [2].

Insect pest management in stored grains has traditionally relied on synthetic insecticides delivered by spraying or fogging, as well as fumigation. However, increasing concerns have led to the search for alternative pest management strategies. These concerns include the demand for agricultural products free of harmful insecticidal residues [3]. Considering these factors, this method focuses on third-instar *P. interpunctella* larvae to investigate their susceptibility to infections by insect-parasitic nematodes.

Entomopathogenic nematodes (EPNs) are obligate insect parasites that invade and kill a wide range of insect hosts. The EPN families Steinernematidae and Heterorhabditidae function through a mutualistic association with their symbiotic bacteria. In Steinernematids, the symbiotic partner belongs to the bacterial genus *Xenorhabdus*.

Previous studies established that *S. carpocapsae* does not rely exclusively on its symbiotic bacteria for pathogenicity. Proteomic analyses identified nematode-derived venom proteins that suppress host immune response and remain lethal even in the symbiotic bacteria’s absence [4]. Consistently, characterization of the serpin-like inhibitor *Sc*-SRP-6 revealed that nematode-encoded immune modulators fine-tune the host defence system and promote successful infection [5].

In *Steinernema* spp*.*, the infective juvenile (IJ) stage is a non-feeding, developmentally arrested, stress-resistant life stage, specialized for host seeking. The IJs actively locate and invade susceptible hosts. Following host death, the IJs resume their development and multiply within the insect cadaver. Eventually, a new cohort of IJs emerges from the nutritionally depleted host and disperses into the soil to locate new hosts [6].

In the present study, two Steinernematid species, *Steinernema carpocapsae* and *S. hermaphroditum,* were used to infect *P. interpunctella* larvae*.* These EPNs differ in their associated symbiotic bacteria which also participate in the infection process. *Steinernema carpocapsae* maintains a mutualistic association with the bacteria *Xenorhabdus nematophila* [7], whereas *S. hermaphroditum* is in an obligate symbiosis with *X. griffiniae* [8]*.* Our previous work has used larvae of the fruit fly *Drosophila melanogaster* to show that *S. carpocapsae* is remarkably more pathogenic than *S. hermaphroditum*. These findings suggest that the *S. carpocapsae*-*X. nematophila* and the *S. hermaphroditum*-*X. griffiniae* complexes may employ distinct infection strategies to infect *D. melanogaster* larvae [9]. Building on these findings, our present study aims to describe an infection method of the natural insect host *P. interpunctella* by two *Steinernema* nematode species and compare their pathogenic competence.

Entomopathogenic nematodes are superb biological control agents for the management of agricultural pests like *P. interpunctella* [10]. While infection of parasitoid wasp-envenomed and non-envenomed *P. interpunctella* larvae with the entomopathogenic nematodes *Heterorhabditis indica* and *Steinernema glaseri* has been reported before [11], infection with other EPNs remains currently unexplored. Our study presents a reproducible infection assay using *P. interpunctella* third-instar larvae to assess susceptibility to two *Steinernema* nematode species under controlled laboratory conditions. Establishing a robust infection method of *P. interpunctella* larvae with *Steinernema* spp. nematodes will lead to a better understanding of EPN-natural insect host interactions and improved biological control applications for agricultural insect pests.

## Method details

*Plodia interpunctella* larval weight and size were not measured in this study. Instead, standardized rearing was achieved by weighing the eggs before they were introduced in the rearing container. Specifically, 10–12 mg of *P. interpunctella* eggs were placed in a weighing boat and transferred to a rearing container containing 45–50 g of wheat bran diet supplemented with 30% glycerol. This approach ensured consistent larval density. The *P. interpunctella* culture was donated by the laboratory of Dr. Arnaud Martin (Department of Biological Sciences, George Washington University), and the larvae were reared under uniform environmental and dietary conditions throughout the experiments. The *S. carpocapsae* and *S. hermaphroditum* nematodes were donated by the laboratories of Dr. Adler Dillman (University of California Riverside) and Dr. John Hawdon (George Washington University School of Medicine and Health Sciences), respectively. Thirty Greiner Bio-One Petri dishes (35 × 10 mm; USA Scientific) were used for each infection assay with the EPNs. Using a sterile spatula, single *P. interpunctella* third-instar larvae were transferred to individual petri dish. Extreme care was taken to make sure that insect larvae were not harmed during their transfer onto the petri dishes. Each infection assay involved ten uninfected larvae as controls, and twenty larvae were exposed to either *S. carpocapsae* or *S. hermaphroditum* IJs. An aliquot of 50 µL of sterile water was added to each uninfected control larva.

For the infection treatments, each *P. interpunctella* larva was exposed to 500 IJs of either *S. carpocapsae* or *S. hermaphroditum* suspended in 50 µL of sterile water. The IJs were subjected to surface-sterilization with 1% bleach prior to their application to the insect larvae. For surface sterilization, *S. carpocapsae* and *S. hermaphroditum* IJs were first suspended in 1 mL of sterile water and transferred to a 1.5 mL microcentrifuge tube. The suspension was centrifuged at 13,000 rpm for 10 s at room temperature to concentrate the IJs into a compact pellet. Following centrifugation, the supernatant was carefully removed. The pellet was then treated with 1 mL of freshly prepared 1% bleach solution and gently mixed to ensure uniform exposure. To eliminate residual bleach, the IJs were washed with 1 mL of sterile water. This washing procedure was repeated a total of five times. After the final wash, the pellet containing the IJs was resuspended in an appropriate volume of sterile water [12]. The nematode suspension was pipetted precisely on top of each *P. interpunctella* larva, ensuring the nematodes were in direct contact with the larval body. The petri dishes containing both the uninfected control larvae and those infected with EPNs were secured using non-airtight laboratory adhesive tape applied around the lid, sufficient to prevent larval escape, while ensuring sufficient airflow, and then placed in a 25 °C incubator under a 12:12 h light:dark photoperiod. All experiments were conducted at 28 °C and at 60–80% relative humidity. *Steinernema* spp. nematodes were observed to enter *P. interpunctella* larvae (Videos 1, 2), which displayed reduced feeding and increased lethargy. Every infection assay was conducted using three biological replicates, with each replicate comprising twenty EPN-infected and ten uninfected control larvae. *Plodia interpunctella* larval survival was monitored and recorded at two-hour intervals. Insect death was defined as a lack of larval reaction to gently poking with a pipette tip. GraphPad Prism software was used to generate Kaplan Meier survival distribution curves and was statistically tested using the Log rank Mantel-Cox test.

## Method validation

**Fig. 1:**
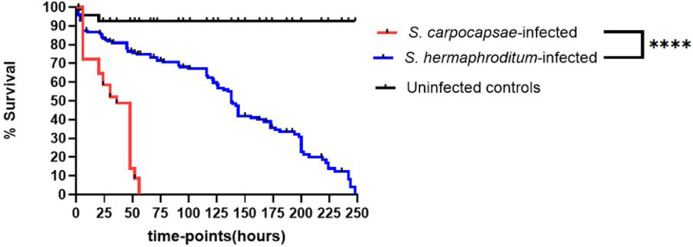
Comparison between the survival of third-instar *Plodia interpunctella* larvae following infection with *Steinernema carpocapsae* or *S. hermaphroditum* infective juveniles. Uninfected control larvae were treated with sterile water only. Larval survival was monitored for 56 h post-infection with *S. carpocapsae* and 248 h following infection with *S. hermaphroditum*. Survival curves were compared using the Log-rank (Mantel-Cox) test in GraphPad Prism. A statistically significant difference(P < 0.0001) was observed between the two infection groups. Survival data represent three independent experiments for each infection group.

**Fig. 2:**
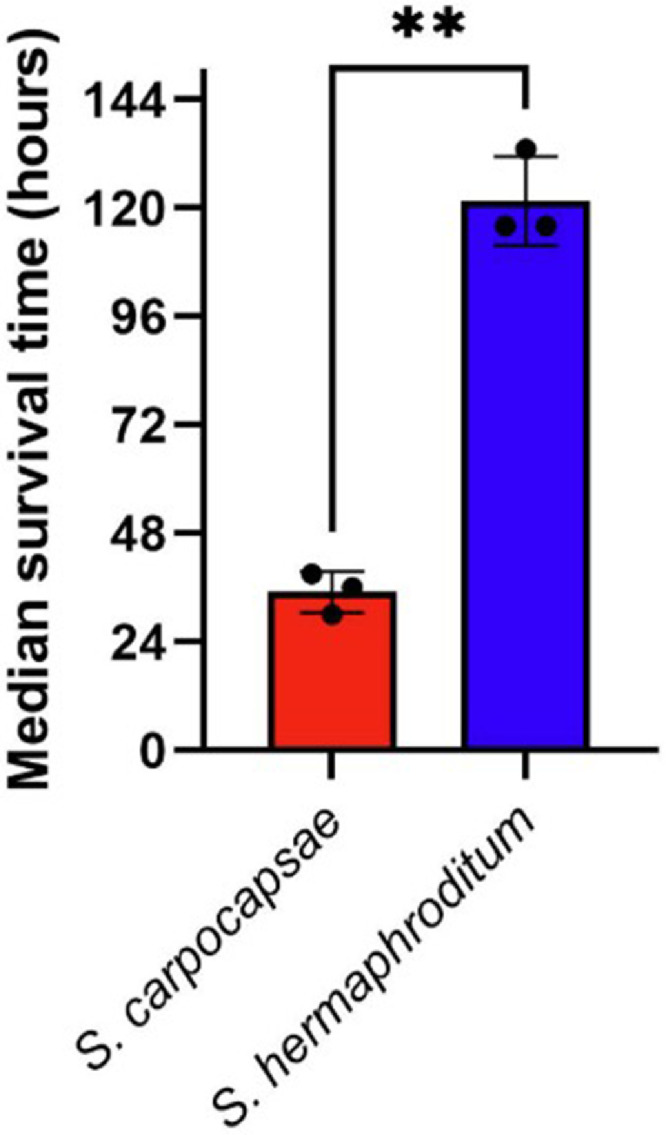
Median survival time of *Plodia interpunctella* larvae infected with either *Steinernema carpocapsae* or *S. hermaphroditum*. Insect larvae were infected with 500 infective juveniles of either *S. carpocapsae* or *S. hermaphroditum*. Data represent three independent experiments, each comprising 20 infected larvae (n = 20 per replicate). Bars indicate median survival time (in hours), with individual data points representing each biological replicate. Error bars show standard deviation. Differences between the treatments were assessed using Welch’s unpaired *t*-test (**p = 0.0011).

Larval survival analysis of *P. interpunctella* third-instar larvae following infection with *S. carpocapsae* and *S. hermaphroditum* IJs confirmed the ability of both the *Steinernema* nematode species to successfully infect and kill the host larvae. Complete (100%) larval mortality was observed at 56 h post-infection with *S. carpocapsae* IJs, while 248 h were required for the *S. hermaphroditum* IJs to kill all larvae (Fig. 1). However, marked differences were observed in the insect larval mortality rate between the two *Steinernema* nematode species. Infection with *S. carpocapsae* resulted in 50% larval mortality which was achieved within 30 h, while *S. hermaphroditum* IJs required 121 h to kill 50% of the exposed larvae (Fig. 2). Comparative analysis between the larval survival curves for the two types of nematode infection revealed that *S. carpocapsae* IJs were significantly more pathogenic than their *S. hermaphroditum* counterparts, inducing faster larval death. The results demonstrate that the *Steinernema* infection protocol for larvae of the natural host *P. interpunctella* is reliable, precise, making it suitable for routine testing of EPN infectivity in other lepidopteran pests.

### Limitations

1. *Plodia interpunctella* culture maintenance: They are highly susceptible to contamination by various pathogens, such as fungi, which can cause high insect mortality. The developmental rate of the insects is sensitive to temperature, humidity, and overcrowding, making it difficult to obtain synchronized larval stages. Changes in the insect diet composition can further complicate insect growth and immune response to infection.
2. Larval fitness: It is important to check carefully the fitness status of the *P. interpunctella* larvae for the infection assays. Larvae of poor quality should not be used in EPN infection experiments because they will lead to misinterpretation of the larval survival results. Moreover, cautious handling is required when transferring insect larvae from the stock culture to the experimental plates, as improper handling may cause stress or physical injury, which will ultimately affect the outcome of the experiment.
3. Delivery of IJs: Handling of the *Steinernema* IJs requires particular attention, as they are easily injured. To facilitate the gentle transfer of the IJs, the tip of the pipette should be trimmed to widen the opening. This would allow sufficient space for approximately 500 IJs suspended in sterile water to be deposited directly onto the *P. interpunctella* larval body.

## Ethics statements

We have used Indian meal moth larvae and entomopathogenic nematodes for our experiments, both of which complied with our institution’s safety and laboratory guidelines.

## CRediT author statement

**Sreeradha Mallick**: Conceptualization, Methodology, Investigation, Validation, Visualization, Writing-original draft, Writing-reviewing and editing. **George Joseph Chakkalakkal**: Conceptualization, Methodology, Investigation, Validation, Visualization, Writing-reviewing and editing. **Taylor Kirby**: Methodology, Investigation, Validation, Visualization, Writing-reviewing and editing. **Juliana Isaac**: Methodology, Investigation, Validation, Visualization, Writing-reviewing and editing. **Vladimir Lažetić**: Supervision, Resources, Conceptualization, Writing-reviewing and editing. **Ioannis Eleftherianos**: Supervision, Funding acquisition, Project administration, Resources, Conceptualization, Writing-reviewing and editing.

## Related research article

S. Mallick, E. Kenney, J. Rashap, I. Eleftherianos, A single entomopathogenic nematode infection assay for *Drosophila melanogaster* larvae, MethodsX 14 (2025) 103,157, doi: 10.1016/j.mex.2025.103157.

## For a published article

None

## Supplementary material *and/or* additional information

None

## Declaration of competing interest

The authors declare that they have no known competing financial interests or personal relationships that could have appeared to influence the work reported in this paper.

## Data Availability

Data will be made available on request.

## Appendix Supplementary materials

Image, video 1 dummy alt text
